# Trait variation and performance across varying levels of drought stress in cultivated sunflower (*Helianthus annuus* L.)

**DOI:** 10.1093/aobpla/plae031

**Published:** 2024-05-27

**Authors:** Ashley M Earley, Kristen M Nolting, Lisa A Donovan, John M Burke

**Affiliations:** Department of Plant Biology, University of Georgia, Athens, GA 30602, USA; Department of Plant Biology, University of Georgia, Athens, GA 30602, USA; Department of Plant Biology, University of Georgia, Athens, GA 30602, USA; Department of Plant Biology, University of Georgia, Athens, GA 30602, USA; The Plant Center, University of Georgia, Athens, GA 30602, USA

**Keywords:** Drought, *Helianthus annuus*, leaf anatomy, plasticity, stomatal density, stomatal size, sunflower, venation

## Abstract

Drought is a major agricultural challenge that is expected to worsen with climate change. A better understanding of drought responses has the potential to inform efforts to breed more tolerant plants. We assessed leaf trait variation and covariation in cultivated sunflower (*Helianthus annuus* L.) in response to water limitation. Plants were grown under four levels of water availability and assessed for environmentally induced plasticity in leaf stomatal and vein traits as well as biomass (performance indicator), mass fractions, leaf area, leaf mass per area, and chlorophyll content. Overall, biomass declined in response to stress; these changes were accompanied by responses in leaf-level traits including decreased leaf area and stomatal size, and increased stomatal and vein density. The magnitude of trait responses increased with stress severity and relative plasticity of smaller-scale leaf anatomical traits was less than that of larger-scale traits related to construction and growth. Across treatments, where phenotypic plasticity was observed, stomatal density was negatively correlated with stomatal size and positively correlated with minor vein density, but the correlations did not hold up within treatments. Four leaf traits previously shown to reflect major axes of variation in a large sunflower diversity panel under well-watered conditions (i.e. stomatal density, stomatal pore length, vein density, and leaf mass per area) predicted a surprisingly large amount of the variation in biomass across treatments, but trait associations with biomass differed within treatments. Additionally, the importance of these traits in predicting variation in biomass is mediated, at least in part, through leaf size. Our results demonstrate the importance of leaf anatomical traits in mediating drought responses in sunflower, and highlight the role that phenotypic plasticity and multi-trait phenotypes can play in predicting productivity under complex abiotic stresses like drought.

## Teaser

Drought is a major agricultural challenge, and its impact is expected to worsen with climate change. To inform the development of more tolerant crops, we examined how leaf traits in cultivated sunflower (*Helianthus annuus* L.) vary in response to four levels of water availability. Overall, biomass declined in response to water limitation; this change was accompanied by decreases in leaf area and stomatal size and increases in stomatal and vein density. The importance of stomatal and vein traits in predicting variation in biomass was mediated, at least in part, through leaf size. Our results demonstrate the influence of leaf anatomical traits on drought responses and highlight the role that phenotypic plasticity and multi-trait phenotypes can play in predicting productivity under drought.

## Introduction

Drought is a major agricultural challenge that limits plant growth and productivity worldwide (Munns 2011). As climate change worsens, droughts are expected to increase in frequency and severity (Foley *et al.* 2011; Munns 2011; IPCC 2023). Moreover, the human population is expected to increase from a current size of 7.6 billion to 9.8 billion by 2050 (United Nations 2017). This poses a challenge as the growing population will increase food demand while much of our agricultural land will be increasingly impacted by environmental stresses including drought. To address this challenge through the development of more resilient crops, we need a better understanding of how traits vary and co-vary in response to drought and how this (co-)variation relates to performance.

Stomatal and hydraulic traits are expected to relate to drought tolerance because they are part of the mechanistic basis for regulating photosynthetic carbon gain and water loss, and operate within a network of leaf economic and allocational traits that determine variation in relative growth rate and performance (i.e. the “flux trait network” hypothesis; Sack *et al.* 2013). Herbaceous crops generally fall at the faster end of the leaf economic spectrum, with higher maximum photosynthesis and leaf nitrogen, lower leaf mass per area, and shorter lifetime (Wright *et al.* 2004; Donovan *et al.* 2014; Reich 2014; Milla *et al.* 2015). Species with a ‘faster’ leaf economic strategy are expected to have a high stomatal conductance to maximize CO_2_ uptake and a high vein density and hydraulic capacity to supply sufficient water for high transpirational water loss (Brodribb *et al.* 2007; Sack *et al.* 2013; Fiorin *et al.* 2016; Harrison *et al.* 2020; Gonzalez-Rebeles *et al.* 2023). As for the mechanisms underlying the trait correlations, they have been hypothesized to result from a combination of selection and different constraints (e.g. biophysical, physiological, genetic, and developmental), but it is important to keep in mind that scale matters when setting up and testing expectations for trait variation and covariation (Donovan *et al.* 2011; Sack *et al.* 2013; Milla *et al.* 2015; Anderegg *et al.* 2018; Agrawal 2020). Here, we focus on stomatal and vein traits as among those of interest for developing drought-tolerant crops (Sack *et al.* 2013; Bertolino *et al.* 2019; Harrison *et al.* 2020; Haworth *et al.* 2021; Hasanuzzaman *et al.* 2023; Petrík *et al.* 2023; Pereira *et al.* 2024; Wang and Chang 2024).

Evidence for the importance of stomatal and vein traits in relation to drought tolerance is available at multiple biological scales. At a broad evolutionary scale, across species or ecotypes of naturally occurring plants, associations between stomatal and vein traits and source environment characteristics are consistent with a potential role for these traits in adaptation to aridity. Using *in situ* sampling and common garden studies, species/ecotypes from more arid habitats have generally been found to have smaller and/or more dense stomata and higher vein density (Uhl and Mosbrugger 1999; Dunlap and Stettler 2001; Dunbar-Co *et al.* 2009; Scoffoni and Sack 2013; Adams *et al.* 2016; Carlson *et al.* 2016; de Boer *et al.* 2016b; Du *et al.* 2021; Yao *et al.* 2021; Xie *et al.* 2022). Additionally, at this broad scale, stomatal density and stomatal size are generally negatively associated, whereas stomatal density and vein length density are generally positively associated (Franks and Beerling 2009; Zhang *et al.* 2012; Scoffoni and Sack 2013; Harrison *et al.* 2020; Haworth *et al.* 2021; Zhao *et al.* 2023; but see Zhang *et al*. 2024). It must, however, be kept in mind that many other traits also differ at this scale, so the adaptive role of an individual trait or trait combination is hard to assess (but see Carlson *et al.* 2016). These studies also demonstrate substantial environmentally induced trait variation (i.e. phenotypic plasticity or acclimation) in the trait-source environment and trait-trait relationships (Dunlap and Stettler 2001; Du *et al.* 2021).

The ability to genetically manipulate plant traits, by creating mutants or altering expression in an existing background, provides another source of evidence for the importance of stomatal and vein traits. In such cases, a lower stomatal density under optimal conditions, accompanied by changes in related traits (e.g. stomatal size, theoretical maximum stomatal conductance, rooting depth, etc.), has generally been associated with higher water use efficiency and/or lower water loss (Yu *et al.* 2008; Doheny-Adams *et al.* 2012; Franks *et al.* 2015; Caine *et al.* 2019; Dunn *et al.* 2019; Li *et al.* 2020; Liu *et al.* 2021; but see Bhaskara *et al.* 2022). Lower stomatal density has also been associated with greater drought tolerance in some instances (e.g. Yu *et al.* 2008; Doheny-Adams *et al.* 2012; Caine *et al.* 2019; Dunn *et al.* 2019; Li *et al.* 2020; Liu *et al.* 2021; Zhao *et al.* 2023), contrasting with the finding of higher stomatal density as a presumed adaptation to drought for plants from arid habitats. Several studies have also reported genetic manipulations of vein length density (e.g. Fambrini *et al.* 2010; Adams *et al.* 2016; Yue *et al.* 2020; Dhakal *et al.* 2021; Haverroth *et al.* 2023), but an association between vein density and drought tolerance has not been established in these types of studies.

The scale most appropriate for breeding is intraspecific, across genotypes or lines, where trait responses and correlations will provide insight into the variation available to breeding efforts. For crop drought responses, plasticity in stomatal and vein traits occurs within a larger and fairly well-known set of general plant responses to water limitation, which include: stomatal closure and associated stomatal limitation of carbon gain, increased non-stomatal limitations of carbon gain, development of newer leaves that are smaller and have a higher LMA, allocational shifts to greater root mass ratio and enhanced root surface area and rooting depth relative to investment in new leaves, osmotic adjustment to maintain turgor and growth as water potentials decline, and less growth and yield (Verslues *et al.* 2006; Munns 2011; Eziz *et al.* 2017). For intraspecific variation in crops and other herbaceous species, acclimation to drought generally results in development of new leaves with higher stomatal density and/or smaller stomatal size, as well as higher vein length density and hydraulic conductance (e.g. Yu *et al.* 2008; Xu and Zhou 2008; Doheny-Adams *et al.* 2012; Sack and Scoffoni 2013; Sun *et al.* 2014; Franks *et al.* 2015; Carlson *et al.* 2016; Shahinnia *et al.* 2016; Ouyang *et al.* 2017; Wang *et al.* 2018; Bresta *et al.* 2018; Lei *et al.* 2018; Dhakal *et al.* 2021; Mano *et al.* 2023; Figure S2), but not necessarily for sunflower (Cardoso *et al.* 2018b; Janzen *et al.* 2023; Tran *et al.*, 2024). There is also some support for the expected negative correlation between stomatal density and stomatal size, as well as positive correlations between stomatal density and vein length density (Doheny-Adams *et al.* 2012; Carins Murphy *et al.* 2014; Sun *et al.* 2014; Shahinnia *et al.* 2016; Xiong *et al.* 2018; Bresta *et al.* 2018; Lei *et al.* 2018; but see Milla *et al.* 2013). When high stomatal conductance is achieved by a high stomatal density and smaller stomata with faster dynamics, it may allow plants to respond more rapidly when water is available (Drake *et al.* 2013; Bertolino *et al.* 2019). Studies that compared the plasticity of traits as they respond (acclimate) to abiotic factors have found no generalities for which types of traits have the highest relative plasticity (Stojnić *et al.* 2015; Eziz *et al.* 2017; Wang *et al.* 2018, 2020; Chen *et al.* 2021; Zhao *et al.* 2023), but greater plasticity in stomatal and vein traits may be associated with greater drought tolerance and/or adaptation to arid habitats (e.g. Adams *et al.* 2016; Bresta *et al.* 2018; Lei *et al.* 2018; Tran *et al.* 2024). Here, we investigate patterns of leaf stomatal and vein responses to varying levels of drought stress in cultivated sunflower (*Helianthus annuus* L.).

Cultivated sunflower is one of the world’s most important oilseed crops (FAO 2018) and an important source of confectionery seeds and ornamental flowers. Because it is often grown on non-irrigated land, sunflower productivity can be largely dependent on natural patterns of precipitation (Meyer *et al.* 2009). While sunflower roots deeply and is able to avoid drought once established, drought remains a major yield-limiting factor across much of the range of production (Connor and Sadras 1992; Hussain *et al.* 2018). A sunflower association mapping population grown under well-watered conditions has shown substantial genetic differentiation for traits, including leaf stomatal and vein traits (Earley *et al.* 2022). In that experiment, genotypes differentiated in multivariate trait space along axes most strongly influenced by leaf traits related to gas exchange, hydraulics, and leaf construction, and stomatal density was negatively correlated with stomatal size and positively correlated with minor vein length density (Earley *et al.* 2022). Cultivated sunflower has been assessed as a moderate drought-tolerant crop, with documented responses including transcriptomic changes, ABA production, osmotic adjustment, high capacitance or buffering against water potential declines, stomatal closure, photosynthesis decline, low minimum transpiration when stomata are closed, xylem cavitation, adjustments of leaf morphology and anatomy, greater relative investment in roots, and reductions in biomass and yield (Stiller and Sperry 2002; Cardoso *et al.* 2018b; Hussain *et al.* 2018; Cardoso *et al.* 2020; Barnhart *et al.* 2022; Janzen *et al.* 2023; Pereira *et al.* 2024; Tran *et al.* 2024). However, Cardoso *et al.* (2018a) found no reduction in sunflower leaf vein length density in response to drought. The continued investigation of sunflower drought tolerance and associated traits remains a vital avenue of research.

The goal of this study is to assess the impact of varying levels of water limitation on leaf anatomical traits and their relationship with overall growth/performance in cultivated sunflowers, using four lines chosen to cover a range of stomatal densities (based on Earley *et al.* 2022). More specifically, we ask the following questions: (i) how do leaf traits vary in response to increasing levels of drought stress, (ii) is trait covariation similar within and across treatments, and (iii) does variation in key leaf traits within and across treatments predict performance across varying levels of drought stress?

## Materials and Methods

### Experimental design

In the summer of 2019, four inbred lines from the sunflower association mapping (SAM) population (Mandel *et al.* 2011) were grown in the Botany Greenhouses at the University of Georgia under a range of watering treatments. These lines correspond to USDA lines RHA 436 (SAM 33) and RHA 364 (SAM 61) and INRA lines SF 076 (SAM 273) and INRA line SF 075 (SAM 282). The experimental design, which included four treatments with four replicates of each genotype in each treatment (*N* = 4 × 4 × 4 = 64 total individuals), were grown under full sun in an unshaded greenhouse in a randomized block design. Following germination, all plants were grown for one week in seedling trays to allow for establishment before being transplanted, one per pot, into 7.6 L plastic pots (HPP200; Haviland Plastic Products, Haviland, OH, USA) filled with a 3:1 mixture of sand and a calcined clay mixture (Turface MVP, Turface Athletics). Greenhouse temperature generally ranged from 82 to 84 °F during the day and 72 to 74 °F at night. Each pot was fertilized with 60 g Osmocote Plus (15-12-9 NPK; Scotts Miracle-Gro, Marysville, OH, USA) and 15 mL each of gypsum (Performance Minerals Corporation, Birmingham, 136 AL) and lime (Austinville Limestone, Austinville, VA, USA) powders for supplemental Ca^2+^. Pots were randomly assigned to one of four treatments: a well-watered control treatment and three drought treatments of varying severity. These treatments were implemented based on the weight of each pot when fully watered. The well-watered control treatment was re-watered daily to 100% of pot capacity while the drought treatments were re-watered to 60%, 40%, and 20% of their capacity based on pot weight. This was done by weighing each pot (with substrate) when fully dry (i.e. substrate before watering and after allowing to sit for three days in the greenhouse) and fully saturated (i.e. pots watered thoroughly and weighed after draining to field capacity to estimate the amount of water required to fully saturate the substrate and determine a target weight for each pot. For context, when converted to gravimetric water content (see below), these treatments correspond to an average of 23.6%, 14.1%, 9.2%, and 4.7% gravimetric water content for the well-watered, mild, moderate, and severe treatments, respectively.


$$\begin{array}{l} Gravimetric\;water\;content\;=\;\;\left(mass\;of\;water/\;mass\;of\;sand\;mixture\right)\;*\;100 \end{array}$$

It should be noted that this method does result in a small overestimation in percent soil moisture. Growth of the plant (increased stored water and dry mass) increased the pot weight, resulting in an overestimation of water remaining in the soil and reducing the amount of water provided in subsequent rewatering. The scale of this effect was, however, minimal due to the large difference in weight between the water in the soil and plant fresh mass. Hereafter, the four treatments will be referred to as the well-watered (100%), mild (60%), moderate (40%), and severe (20%) drought treatments. Following transplantation and initial saturation of the soil, the plants were allowed to acclimate for three days before the treatments commenced. Once the treatments started, pots were re-weighed daily between 9:00 and 11:00 AM using a postage scale (PS-IN202; Prime Scales, Chino, CA, USA) and allowed to dry to their target levels. Generally, pots within each treatment reached target levels on approximately the same date. Once they dipped below their target weight, pots were individually re-watered to bring them back to their target weight. Note that individuals in the mild treatment reached their target level sooner than individuals in the moderate treatment. While individuals in the severe treatment experienced the most severe drought, most did not reach their target level before the end of the experiment, and all of them lagged behind individuals in the moderate treatment. Importantly, however, we observed a significant treatment effect with an ordered reduction in biomass with increasing drought severity (see Results, below). See Supporting Information—Fig. S1 provides water content data for all individual pots across treatments and genotypes for the duration of the experiment. The experiment was allowed to run for 24 days following implementation of the drought treatments at which time all plants were harvested.

At harvest, traits were measured as described by Earley *et al*. (2022). Briefly, on harvest day, the two most recent fully expanded leaves were collected for analysis. Based on a comparable study that marked and followed *H. annuus* leaf growth (Tran 2021), these leaves were likely leaf buds at the start of the drought, with a substantial portion of subsequent leaf cell division and expansion taking place as drought developed. One of the most recently fully expanded leaves was designated for estimation of leaf mass per area (see Table 1 for a complete list of traits, functional groupings, abbreviations used in figures, and units) and the other was designated for anatomical characterization. Immediately prior to leaf harvest, chlorophyll content index was measured (Apogee MC-100) on the anatomical leaf for each plant. Following harvest, the leaf designated for leaf mass per area was scanned on a flatbed scanner to determine leaf area, dried, and weighed to determine the dry biomass of the lamina and midrib (excluding the petiole). Leaf mass per area was calculated as ((leaf dry mass)/(leaf area)). Leaf midrib density was calculated as ((midrib dry mass)/(midrib volume)), with midrib volume estimated as a cone shape, and the width of the midrib base as the cone diameter. Midrib mass fraction was calculated as ((midrib mass)/(whole leaf mass)). All remaining biomass was likewise collected, dried, and weighed to determine root, stem, leaf, and flower bud biomass, which were summed into aboveground biomass and total biomass. Note that flower bud biomass was not used in further analyses because only a subset of plants had produced flower buds at the time of sampling. Stem mass fraction, leaf mass fraction, and root mass fraction were calculated by dividing each biomass component by total biomass.

**Table 1.** List of traits, abbreviations, units, and functional groupings.

	Trait abbreviation	Full trait name	Units
Whole plant growth	AG_Bio	Aboveground biomass	g
	Total_Bio	Total biomass	g
Whole plant biomass allocation	Leaf_MF	Leaf mass fraction	gleaf/gplant
	Stem_MF	Stem mass fraction	gstem/gplant
	Root_MF	Root mass fraction	groot/gplant
Leaf economics spectrum (LES)	Leaf_Area	Individual leaf area	m^2^
	LMA	Leaf mass per area	g/m^2^
Stomatal traits	SD_Bot	Stomatal density, bottom of leaf	stomata/mm^2^
	SD_Top	Stomatal density, top of leaf	stomata/mm^2^
	SD_Avg	Stomatal density, average of top and bottom	stomata/mm^2^
	SPL_Bot	Stomatal pore length, bottom of leaf	µm
	SPL_Top	Stomatal pore length, top of leaf	µm
	SPL_Avg	Stomatal pore length, average of top and bottom	µm
	SGCW_Bot	Stomatal guard cell width, bottom of leaf	µm
	SGCW_Top	Stomatal guard cell width, top of leaf	µm
	SGCW_Avg	Stomatal guard cell width, average of top and bottom	µm
	Stomatal_Ratio	Stomatal ratio (bottom of leaf/total)	Unitless
	Total_Stomata	Total number stomata, sum of top and bottom	stomatal count
Leaf venation	Minor_VLA	Minor vein length per area, or minor vein density	mm/mm^2^
	Second_VLA	Secondary vein length per area, or secondary vein density	mm/mm^2^
	Major_VLA	Major vein length per area or major vein density (includes secondary veins and midrib)	mm/mm^2^
	SV	Stomata number per vein length	stomata/mm
	Midrib_Density	Midrib density	mg/cm^2^
	Midrib_MF	Midrib mass fraction	gmidrib/gleaf
Gas exchange physiology	Chlorophyll	Leaf chlorophyll content index	Unitless
	Gsmax	Maximum theoretical stomatal conductance	mol/m^2^*s

The anatomy leaf was cut in half lengthwise along the midrib and one half was dried and stored. This half was rehydrated overnight in water at a later date and the adaxial and abaxial (hereafter top and bottom) surfaces were pressed into dental putty (President Dental Putty; Coltène/Whaledent Inc., Cuyahoga Falls, OH, USA) to produce an impression of the epidermis that could be used to visualize and analyze stomatal traits following the general methods of Weyers and Johansen (1985). Note that the use of rehydrated leaves may have led to a slight overestimation of stomatal and vein length density due to a small amount of shrinkage compared to fresh leaves (Blonder *et al.* 2012; de Boer *et al.* 2016b), but effects were assumed to be equivalent across individuals within this study. Nail polish was applied to each impression and lifted off with tape and imaged; this was done separately for the top and bottom surfaces of each leaf (Hilu and Randall 1984; Weyers and Johansen 1985). Imaging for stomatal density and size involved taking images of both the top and bottom impressions for stomatal density and size measurements (5× and 100×, respectively) as in Earley *et al.* (2022). Stomatal density was determined for the top and bottom of leaves (i.e. the adaxial and abaxial surfaces). Average stomatal density was calculated as (((stomatal density top) + (stomatal density bottom))/2). Stomatal pore length was determined, along the longest pore axis, for the top and bottom of leaves. The average pore length was calculated as (((stomatal pore length top) + (stomatal pore length bottom))/2). Stomatal guard cell width was used as an estimate of stomatal pore depth for the top and bottom of leaves. The average guard cell width was calculated as (((stomatal guard cell width top) + (stomatal guard cell width bottom))/2). The total number of stomata was calculated as (((stomatal density top) + (stomatal density bottom)) * leaf area). Stomatal ratio was calculated as ((stomatal density bottom)/((stomatal density top) + (stomatal density bottom))). Maximum stomatal conductance (*g*_smax_), the theoretical maximum rate of gas exchange if all stomata were fully open, was calculated based on stomatal density and size measurements using the approach of (Dow *et al.* 2014).

The second half of the anatomy leaf was stored in formalin-acetic acid-alcohol (FAA) fixative for imaging and analysis of vein traits. Each sample was cleared, stained, and imaged as described in Earley *et al.* (2022). This included both scanning on a flatbed scanner and imaging tran four different fields of view under a microscope at 5×. Second-order vein length was measured from the scanned images by manually tracing veins that branched off of the midrib (i.e. primary vein). Secondary vein length per area or ‘secondary vein density’ was calculated as ((second order vein length)/(leaf area)). Major vein length per area or ‘major vein density’ was calculated as ((midrib length) + (second order vein length))/(leaf area)). Minor vein length per area or ‘minor vein density’, representing all veins not included in major vein density, was calculated as (length of minor veins)/(leaf area)) with the length of minor veins estimated from the microscope images using a deep neural network as described by Earley *et al*. (2022)). Finally, a composite trait of stomata number per vein length was calculated as ((stomatal density average)/ (minor vein length per area) (Zhao *et al.* 2017).

### Data analysis

All data analyses were conducted using R v43.24.13 (R Core Team 2021) in R Studio v1.3.1093 (RStudio Team 2015).

#### Trait responses to increasing levels of drought stress.

Initially, a two-way ANOVA with genotype, treatment, and their interaction as the main effects was used to test for variation among genotypes and treatments and to calculate estimated marginal trait means (in the emmeans package; Lenth 2020) across replicates for each genotype after removing block effects (i.e. block included as a random intercept). In cases where the fitted mixed effects model neared singularity, the random block effect was dropped from the model and a linear model was implemented, following the recommendations from Barr *et al.* (2013). When a significant treatment effect was detected, trait differences between treatment levels were determined using post hoc Tukey’s tests (in the emmeans package; Lenth 2020). All ANOVA models were run using rank-ordered trait data, given the presence of a few data outliers driving non-normality of residuals when the models were run using the raw data. It is worth noting that other common types of data transformation (e.g. log transformation and square-root transformation) did not significantly improve model fit, and thus we present the results using the rank-ordered data as a more conservative approach.

As a way to standardize and visualize the plasticity of each trait in our dataset, we calculated a relative distance plasticity index (RDPI) of each genotype using the estimated marginal mean values. RDPI, which was calculated as described by Valladares *et al.* (2006), but modified to range from −1 to + 1 to show the direction of change, allows for cross-treatment comparisons and is strongly correlated with the majority of other measures of phenotypic plasticity. It was calculated for each trait and treatment versus the well-watered control, as follows: RDPI = (mean stress—mean well-watered)/ (mean stress + mean well-watered).

#### Trait covariation within and across treatments.

 Individual plant-level data were used to estimate bivariate trait correlations and to create correlation matrices across all treatments and per treatment using the R package *corrplot* (Wei and Simko 2021). Mantel tests were performed using correlation matrix data and the R package *vegan* to compare correlation matrices and test for differences among treatments (Oksanen *et al.* 2013).

Principal component analysis (PCA) was conducted using the function prcomp() and the package *ggfortify* (Tang *et al.* 2016; Horikoshi and Tang 2018) to visualize the multivariate trait correlations. PCAs were done with all traits listed in Table 1, except using averages for stomatal stomatal density, stomatal pore length, and stomatal guard cell width. A PCA was performed combining data from all treatments, and for this PCA differences between treatments were investigated using Hotellings *t*^*2*^ test on the first two principal components. All graphs were made using the R package *ggplot2* (Wickham 2016). PCAs were then performed for each treatment separately.

#### Associations of trait variation with performance.

 A linear mixed effects regression model using the Bayesian statistical package *brms* (Bürkner 2017, 2018, 2021) was used to evaluate the relationship between trait variation and variation in performance within each treatment with block and genotype included as random intercepts to account for non-independence amongst replicates. We chose to run these regression models in a Bayesian framework as this approach is less limited by small sample sizes compared to the number of parameters to be estimated (Hobbs and Hooten 2015). Given the highly correlated nature of many of the traits in our dataset, we selected four leaf traits that reflect important axes of leaf trait variation in cultivated sunflowers (see Earley *et al.* 2022): average stomatal pore length and average stomatal density (important structural traits that influence gas exchange; Lawson and Blatt 2014), minor vein length per area (a venation trait related to leaf water transport; Sack and Scoffoni 2013), and leaf mass per area (which reflects the investment in biomass and leaf construction; Wright *et al.* 2004). We centered and scaled all traits to have a mean of zero and a standard deviation of one. Due to a few extreme values for leaf mass per area, we natural-log transformed this trait before centering and scaling to improve model fit. The model included the four leaf traits and the interaction of each with stress level as main effects. As noted above, block and genotype ID were included as random intercepts in the model. Default priors were used (Bürkner 2017, 2018, 2021) with a step size of 0.99, four chains with 2000 iterations each (with 1000 iterations for a warm-up), and no thinning for a total of 4000 samples from the posterior. The model was evaluated for efficient mixing. No Rhat values greater than 1.00 were reported and there were no divergent transitions. The treatment level-specific coefficients relating each leaf trait to total biomass are reported below, as are the Bayesian marginal *R*^2^ estimates (Gelman *et al.* 2019) which estimate the variance explained by the fixed effects in the model.

## Results

### Leaf trait variation in response to increasing levels of drought stress

**Figure 1.**
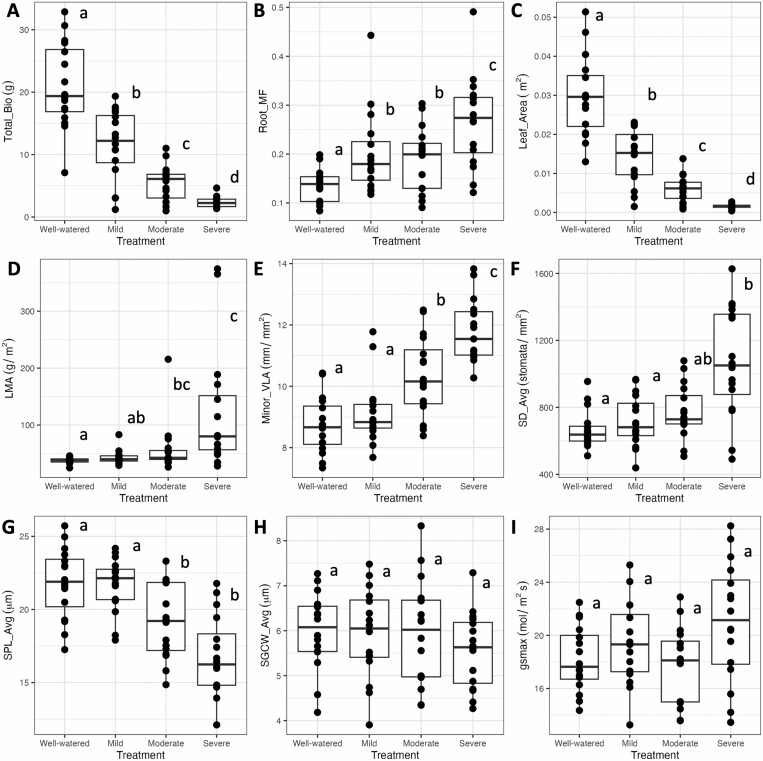
Boxplots of individual variation in representative traits across treatments. Traits of interest include (A) total biomass (Total_Bio), (B) root mass fraction (Root_MF), (C) leaf area (Leaf_Area), (D) leaf mass per area (LMA), (E) minor vein length per area (Minor_VLA), (F) average stomatal density (SD_AVG), (G) average stomatal pore length (SPL_AVG), (H) average stomatal guard cell width (SGCW_AVG), and (I) theoretical maximum stomatal conductance (*g*_smax_). For each trait, letters indicate significant differences across treatments based on nonparametric post hoc contrasts.

Treatment effects were significant for many leaf anatomical and higher-level (i.e. whole plant) traits (Tables 2, **Supporting Information—Table**S1; also see Table 1 for traits abbreviations and units). As expected, increasing drought severity reduced plant growth, with total biomass exhibiting a 37%, 69%, and 88% decline in the mild, moderate, and severe treatments, respectively (Fig. 1). For biomass allocation, leaf mass fraction decreased in response to drought, while root mass fraction increased. For the most recently fully expanded leaf at harvest, leaf area decreased, and leaf mass per area increased.

**Table 2.**
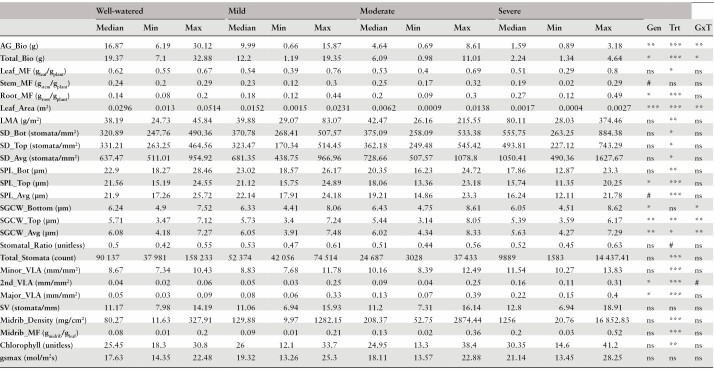
List of all traits measured along with the median and range of trait values. Significance from the ANOVA on ranked trait values indicated as (***P ≤ 0.001, **P ≤ 0.01, *P ≤ 0.05, #*P* ≤ 0.1, ns = not significant) and adjusted *R*^2^ from the model are also presented. Trait abbreviations follow Table 1. Gen = genotype; Trt = treatment; GxT = genotype-by-treatment interaction.

For stomatal traits, stomatal density (top, bottom, and average) increased with treatment severity, while stomatal size (stomatal pore length top, bottom, and average; stomatal guard cell width top and average)) and total number of stomata decreased. Stomatal ratio and stomatal number per vein length did not change in response to drought (Table 2, Fig. 1). For venation traits (e.g. minor, major, and secondary vein length per area) there was a general pattern whereby traits increased with treatment severity, as did midrib density and midrib mass fraction, but the number of stomata per vein length was not affected. For the gas exchange-related traits, chlorophyll content generally increased with treatment severity, while *g*_*smax*_ was not consistently affected. Genotypic effects were also significant for a number of traits and there also was evidence of genotype-by-treatment interactions for several traits (Table 2).

**Figure 2.**
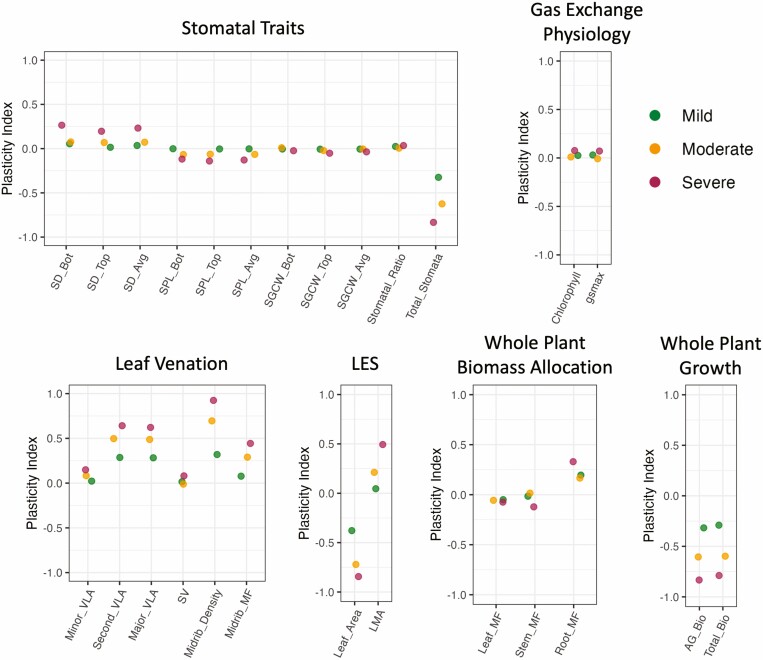
Plasticity for each trait (as relative distance plasticity index, RDPI), calculated for each treatment versus the well-watered control. Points indicate the average estimated marginal mean for each trait for each treatment. Trait abbreviations and functional groupings follow Table 1.

We additionally looked at the trait responses estimated as relative plasticity (RDPI) where values can range −1 to +1 with positive or negative values reflecting increases or decreases in trait values in response to the treatment, and 0 representing no plasticity. Trait plasticity increased with increasing drought severity for most traits and was consistent across genotypes (Fig. 2). In general, smaller-scale leaf anatomical traits such as average stomatal density, average stomatal pore length, and minor vein length per area were less plastic than larger-scale traits related to construction and growth, such as secondary and major vein length per area, leaf area, aboveground biomass and total biomass.

### Trait covariation across and within treatments

#### Across treatments.

**Figure 3.**
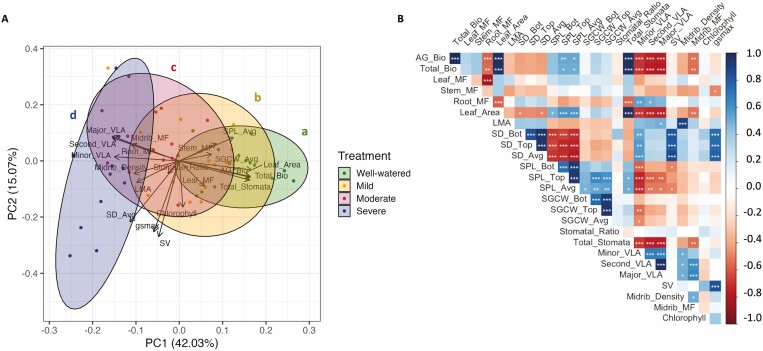
(A) Principal component analysis (PCA) of all traits in Table 1 (abbreviations follow Table 1) except using averages for stomatal density, stomatal pore length, and stomatal guard cell width. Colors indicate treatments and lower-case letters associated with each of the colored ellipses (which reflect the 95% confidence intervals) indicate the results of Hotelling’s *t*^2^ tests for differences between treatments. *P*-values were adjusted for multiple comparisons using a Bonferroni correction. (B) Bivariate correlation matrix of all traits in Table 1 across all treatments. Values were calculated using individual plant-level data and significance tests were corrected for multiple comparisons using a Bonferroni correction. Positive correlations are in blue and negative correlations are in red. Shading gives a relative indication of the magnitude of the estimate. ***P ≤ 0.001, **P ≤ 0.01, * *P* ≤ 0.05.

Looking at trait relationships across treatments meant that there was an increased range of trait variation being assessed, by combining the genotypic differentiation within each treatment and the environmentally induced plasticity in response to water limitations. At this scale across treatments, the bivariate correlations (Fig. 3B) showed that total biomass was positively correlated with aboveground biomass, leaf area, and total number of stomata, but total biomass was negatively correlated with minor, secondary, and major vein length per area. Leaf area was negatively correlated with average stomatal density, average stomatal pore length, and minor vein length per area. Average stomatal density was negatively correlated with average stomatal pore length, positively correlated with minor vein length per area, and showed no detectable correlation with average stomatal guard cell width. Average stomatal pore length was positively correlated with average stomatal guard cell width. A corresponding PCA was used to visualize the plant multivariate phenotypes across all treatments (Fig. 3A). PC1 and PC2 explained 42.0% and 15.1% of the variation, respectively. PC1 was dominated by traits related to plant size (total biomass, aboveground biomass, leaf area, and total number of stomata) and vein traits (Table 3). PC2 was dominated by traits related to stomata and veins (stomatal number per vein length, *g*_smax_, and average stomatal density). PC3 was dominated by traits related to construction costs and allocation (leaf mass fraction, midrib density, leaf mass per area, and root mass fraction). The responses in multivariate space were largely consistent with bivariate trait responses already presented, with average stomatal density and average stomatal pore length being negatively correlated, and average stomatal density and minor vein length per area being positively correlated.

**Table 3.**
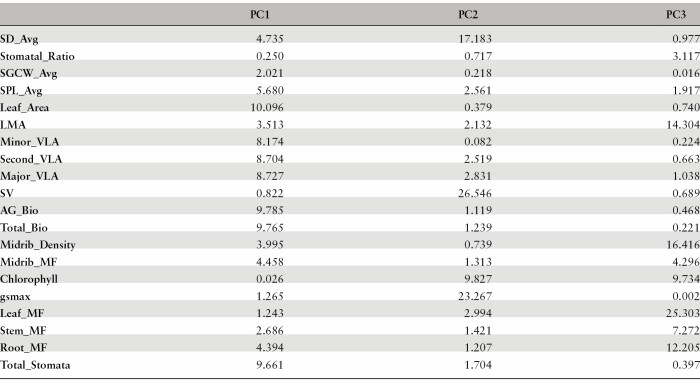
Trait loadings (percentage of trait variation explained by each trait in the associated principal component [PC]) for each of the first three PCs in Fig 3A. Trait abbreviations follow Table 1.

#### Within treatments.

**Figure 4.**
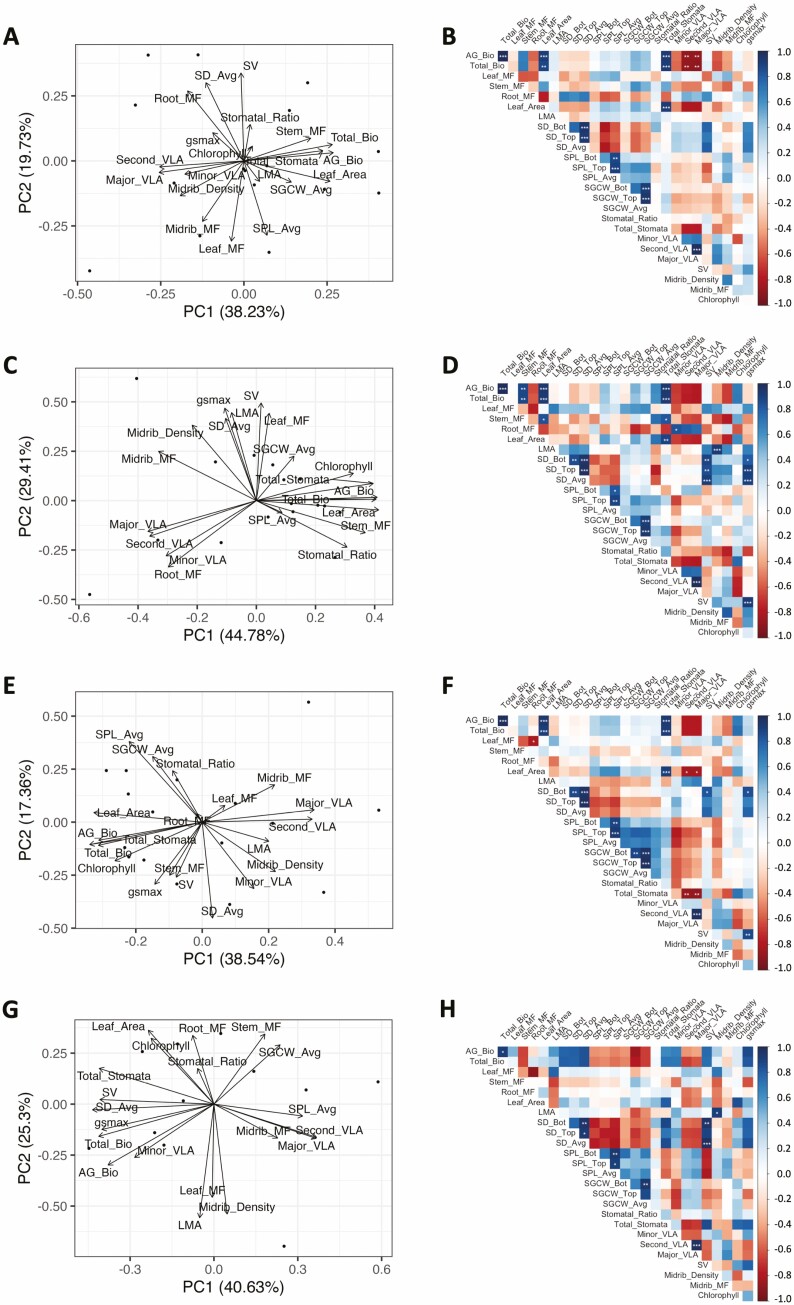
(A, C, E, G) Principal component analysis (PCA) of all traits in Table 1, except using only averages for stomatal density, stomatal pore length, and stomatal guard cell width. Well-watered, mild, moderate, and severe drought treatments, respectively. (B, D, F, H) Bivariate correlation matrices for all traits in Table 1 for well-watered, mild, moderate, and severe drought treatments, respectively. Values were calculated using individual plant-level data and significance tests were corrected for multiple comparisons using a Bonferroni correction. Positive correlations are in blue and negative correlations are in red. Shading gives a relative indication of the magnitude of the estimate. ***P ≤ 0.001, **P ≤ 0.01, * *P* ≤ 0.05.

The bivariate trait relationships within treatments are presented in Fig. 4B, D, F, and H for the well-watered, mild, moderate, and severe treatments, respectively. Mantel tests indicated that none of the treatment matrices differed significantly from one another (i.e. the null hypothesis of a lack of correlation between matrices was rejected [*P* < 0.001] for all comparisons), although this result should be interpreted with caution due to non-independence amongst replicates and a limited sample size. Within most treatments, total biomass was positively correlated with aboveground biomass, leaf area, and total number of stomata, whereas total biomass tended to be negatively correlated with secondary and major vein length per area. There was a trend for average stomatal density and average stomatal pore length to be negatively correlated in each treatment, but the correlations were not significant. There were no significant correlations or consistent trends for average stomatal density and minor vein length per area within treatments.

A corresponding principal component analysis was used to visualize the plant multivariate phenotypes within each treatment. The top two principal components axes (i.e. PC1 + PC2) accounted for 58.0%, 74.2%, 55.9%, and 65.9% of the observed trait variation in the well-watered, mild, moderate, and severe treatments (Fig. 4A, C, E, and G, respectively). The traits dominating PC1 and PC2 varied by treatment but, in general, were biomass or closely related traits (see Supporting Information—Table S2A–D). The PCA plots visually reinforce that there was a trend for average stomatal density and average stomatal pore length to be negatively correlated in each treatment, but that there was no consistent trend for average stomatal density and minor vein length per area.

### Relationship between trait variation and performance

**Figure 5.**
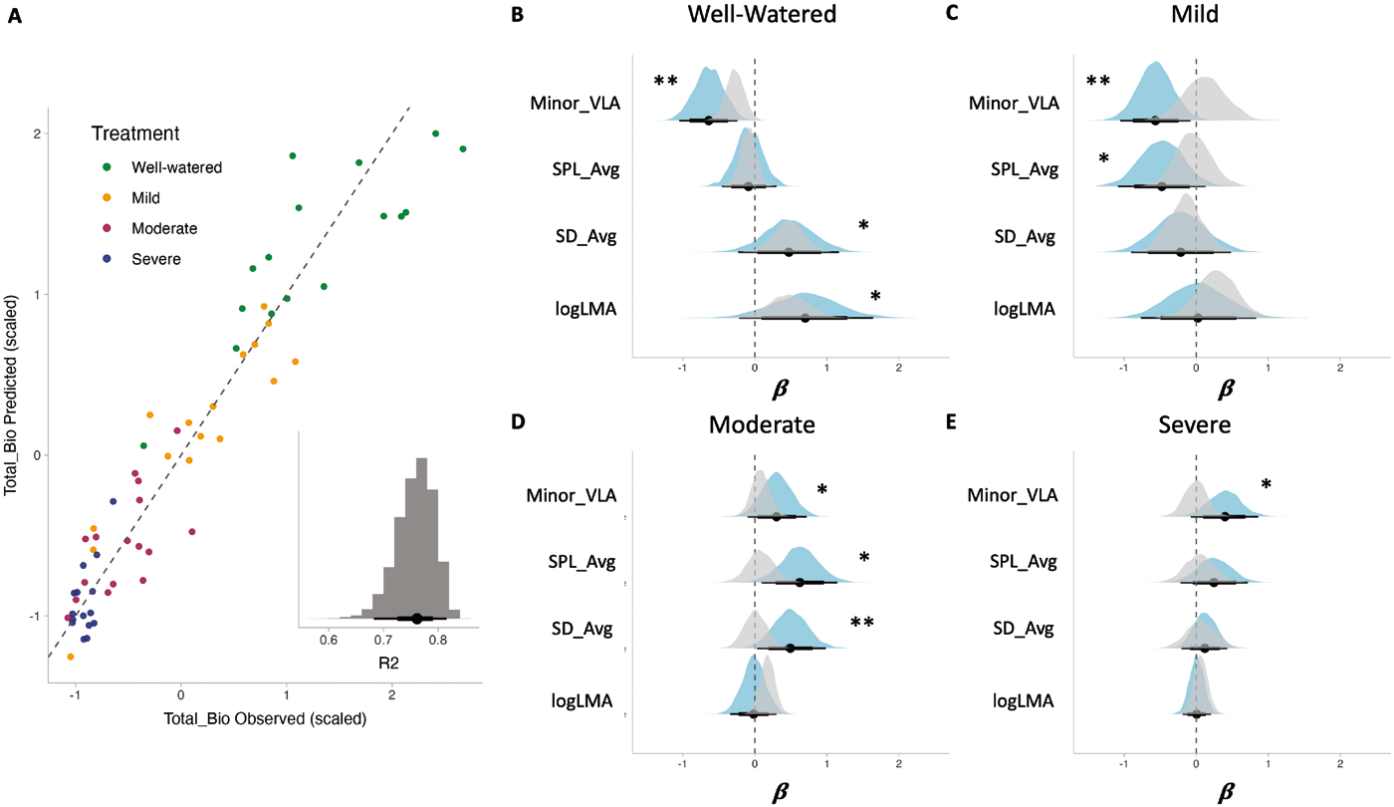
Results of our analysis of performance as a function of variation in minor vein length per area (Minor_VLA), average stomatal pore length (SPL_Avg), average stomatal density (SD_Avg), and leaf mass per area (LMA, natural log-transformed) across treatments using a linear mixed effects regression model. (A) Predicted versus observed total biomass (Total_Bio, centered with a mean of zero and scaled to a standard deviation of one), with colors corresponding to different treatment levels. Inset histogram of the estimated Bayesian *R*^*2*^, for fixed effects only (full posterior distribution of the model presented, with the black dot indicating the mean of the distribution, the thin black line the 95% CI, and the heavier black bar the 80% CI). (B) The mean estimated association between each trait and total biomass (i.e., *β*), under well-watered conditions. (C) The mean estimated association between each trait and total biomass (i.e., *β*), under mild conditions. (D) The mean estimated association between each trait and total biomass (i.e., *β*), under moderate conditions. (E) The mean estimated association between each trait and total biomass (i.e., *β*), under severe conditions. In (B)–(E) all output reflects posterior summaries from the model. Blue distributions indicate the associations estimated in the model that does not include leaf area, with the black dot indicating the estimated mean value. The heavier bars indicate the 80% CIs for each estimate, and the lighter lines indicate the 95% CIs. Estimates with 95% CIs not overlapping zero were interpreted as having strong support (**), and those with 80% CIs not overlapping zero with moderate support (*). The gray distributions indicate the associations estimated in the model that includes natural log-transformed leaf area as an additional covariate in the model (see Supporting Information—Table S3 and S4 for full model summaries).

The four key traits (minor vein length per area, average stomatal pore length, average stomatal density, and leaf mass per area), selected to reflect important functional axes of leaf trait variation in cultivated sunflowers (Earley *et al.* 2022), exhibited strong predictive power overall for explaining variation in plant performance (total biomass) across treatments with an overall model marginal *R*^2^ = 0.78 (Fig. 5A, see Supporting Information—Table S3). Within each treatment traits showed both positive and negative associations with plant performance, but the sign of the relationships between traits and performance often varied by treatment (Fig. 5B–E, [see Supporting Information—Table S3; estimates with 95% CIs not overlapping zero were interpreted as having the strong support of the association, and those with non-overlapping 80% CIs with moderate support, see Materials and Methods). Minor vein length per area was negatively associated with total biomass in the well-watered control (*B*_Minor_VLA_well-watered_ = −0.64, 95% CI: [−1.05, −0.25]) and mild drought treatments (*B*_Minor_VLA_mild_ = −0.57, 95% CI: [−1.05, −0.08]), but positively associated under moderate drought (*B*_Minor_VLA_moderate_ = 0.30, 80% CI: [0.04, 0.57]; note this was not significant at the 95% CI) and severe drought treatments (*B*_Minor_VLA_severe_ = 0.40, 80% CI: [0.10, 0.68]; note that this was not significant at the 95% CI). Average stomatal density had a positive association with total biomass in the well-watered control (*B*_SD_Avg_well-watered_ = 0.47, 80% CI: [0.04, 0.92]; note this was not significant at the 95% CI) and moderate drought treatments (*B*_SD_Avg_moderate_ = 0.49, 95% CI: [0.03, 0.98]), but no association was detected in the mild and severe drought treatments. Average stomatal pore length was negatively associated with total biomass under the mild drought treatment (*B*_SPL_Avg_mild_ = −0.48, 80% CI: −0.86, −0.09]; note this was not significant at the 95% CI) but positively associated under the moderate drought treatment (*B*_SPL_Avg_moderate_ = 0.62, 95% CI: [0.10, 1.15]). Finally, log-transformed leaf mass per area had a positive association with total biomass under well-watered conditions (*B*_logLMA_well-watered_ = 0.70, 80% CI: [0.09, 1.28]; note this was not significant at the 95% CI) but no clear association under mild, moderate, or severe drought conditions.

The importance of considering traits in a multivariate manner became even more clear when we investigated the influence of leaf size in our dataset—recall that leaf area is the size of a single, fully expanded leaf. From our bivariate and multivariate investigations of trait correlations, we saw that leaf size covaries strongly with plant size traits (aboveground biomass and total biomass) and with many anatomical traits, especially minor vein length per area under some treatments (see above). To formally investigate the role leaf size plays in mediating the associations between leaf trait variation and variation in plant performance, we ran an additional model in which we added leaf area (natural log transformed) along with the above four focal traits and including block and genotype as random intercepts, as above. The overall *R*^2^ for this model is even higher than observed with the four trait model (model marginal *R*^2^ = 0.94, not shown) and in a formal model comparison the model that includes leaf area is preferred, despite the model being more complex (see see Supporting Information—Table S5). Notably, inclusion of leaf area in the model influences the predicted associations between variation in our four focal traits and variation in total biomass (Fig. 5, panels B–E gray distributions). For example, under well-watered conditions the strength of the negative association between minor vein length per area and total biomass is over double the magnitude in the model without leaf area compared with the model that includes leaf area (i.e. we detect a much stronger negative association between minor vein length per area and total biomass when not accounting for the effects of leaf area). The associations between average stomatal pore length, average stomatal density, log-leaf mass per area, and total biomass under well-watered conditions are much less affected. In the remaining three treatments, almost all trait associations with biomass become smaller in magnitude when leaf area is included in the model, and in most instances become non-significant under the 80% CI (**see Supporting Information—**Tables S3, S4).

## Discussion

Drought is a major challenge that reduces plant growth and limits crop productivity worldwide. Here we investigated drought in cultivated sunflower with a range of water limitation treatments that resulted in biomass reductions, a decline of 37%, 69%, and 88% in the mild, moderate, and severe treatments, respectively. The larger declines in our study are in the range that likely would result in total crop loss if drought levels were sustained, but might be recoverable if water was limited for a relatively short interval during vegetative growth and again became available (Janzen *et al.* 2023). With the biomass decline, leaf mass fraction decreased and root mass fraction increased as is commonly observed for drought responses, although there is still a debate in the literature as to the extent that the allocational shift in response to resource limitation are passive allometric changes or active adjustments associated with optimal partitioning for capturing the most limiting resource (Poorter *et al.* 2012; Eziz *et al.* 2017; Wang *et al.* 2020). Either way, the plants in our study adjusted biomass and biomass allocation as generally expected for water-limited herbaceous plants.

### Leaf traits vary in response to increasing levels of drought stress

At the leaf level, we examined the traits of the most recently expanded leaf pair for each plant. These leaves were emerging buds at the beginning of the treatments and developed as the drought progressed. Thus, this is a conservative assessment of responses to drought, since the responses might have been more extreme if we had examined leaves fully produced after the target soil moisture limitations were reached. That was not possible in this experiment because we started the treatments (i.e. withholding water from the mild, moderate, and severe water limitation treatments) at an early vegetative stage (ca. 2–4 leaf stage), and harvested all plants when a portion of the plants initiated flower bud production.

Overall, as expected, the leaf area of individual leaves decreased in response to drought and leaf mass per area increased. For stomatal traits, average stomatal density increased and average stomatal pore length decreased consistent with other responses to drought (Yu *et al.* 2008; Sack and Scoffoni 2013; Sun *et al.* 2014; Franks *et al.* 2015; Carlson *et al.* 2016; Ouyang *et al.* 2017; Bresta *et al.* 2018; Lei *et al.* 2018; Mano *et al.* 2023). However, other studies, including several for sunflower, documented no consistent effect on stomatal density (Xu and Zhou 2008; Doheny-Adams *et al.* 2012; Sack and Scoffoni 2013; Shahinnia *et al.* 2016; Cardoso *et al.* 2018b; Tran *et al.* 2024; Fig. S2) or a decline in both stomatal size and density (Janzen *et al.* 2023). It may be that the more important trait is the composite trait of theoretical *g*_smax_ (calculated from stomatal density and pore length and depth; Bertolino *et al.* 2019), but there were inconsistencies among studies in that response as well. While we found no consistent decline in *g*_smax_ in response to drought, it has previously been shown to decline in response to drought in sunflower and other species (Lei *et al.* 2018; Janzen *et al.* 2023; Tran *et al.* 2024). The apparent discrepancy between sunflower studies may be due to differences in genotypes used or to differences in the logistics of drought implementation relative to leaf development (e.g. timing, duration, and maximum stress imposed). At the very least, this suggests that there is substantial plasticity in stomatal density and size in response to drought that is available for exploration in relation to drought tolerance.

Minor vein length per area increased in response to drought, consistent with phenotypic responses to drought in some other herbaceous species (Lei *et al.* 2018; Wang *et al.* 2018; Dhakal *et al.* 2021; Mano *et al.* 2023). An increase in vein density is likely associated with greater hydraulic conductance and the maintenance of hydraulic function may be critical for plant survival under drought (Yao *et al.* 2021). However, our results are not consistent with those of Cardoso *et al*. (2018) who found that sunflower leaves produced during a drought did not alter their vein density. Again, this may be due to differences in genotypes or experimental logistics and deserves further exploration.

The phenotypic response to drought of increased stomatal density and vein density in this study is in the same direction as the evolutionary scale genetic differentiation of traits (Strobel and Sundberg 1983; e.g. Uhl and Mosbrugger 1999; Dunlap and Stettler 2001; Dunbar-Co *et al.* 2009; Drake *et al.* 2013; Sack and Scoffoni 2013; Adams *et al.* 2016; Carlson *et al.* 2016; de Boer *et al.* 2016a; Du *et al.* 2021; Yao *et al.* 2021; Xie *et al.* 2022), wherein individuals from increasingly dry habitats tend to exhibit smaller, denser stomata, and/or an associated increase in vein density. Thus, the plastic responses of leaf anatomical traits in our study parallel putatively adaptive solutions to water limitation that have evolved within and among plant species. It has been argued that smaller stomata are able to close more quickly, so that this phenotypic response to stress might improve the ability of plants to rapidly adjust stomatal conductance in response to changes in water availability (e.g. Brodribb *et al.* 2007; Sack and Scoffoni 2013; Bertolino *et al.* 2019). However, these results appear to conflict with the results of genetically manipulating plants to reduce stomatal density (among other trait changes) which resulted in increased drought tolerance (Yu *et al.* 2008; Doheny-Adams *et al.* 2012; Caine *et al.* 2019; Dunn *et al.* 2019; Li *et al.* 2020; Liu *et al.* 2021; Zhao *et al.* 2022). This suggests that the consensus that stomatal and vein traits are likely to be important for breeding more water-use-efficient and drought-tolerant crops (Sack *et al.* 2013; Bertolino *et al.* 2019; Harrison *et al.* 2020; Haworth *et al.* 2021; Hasanuzzaman *et al.* 2023; Petrík *et al.* 2023; Pereira *et al.* 2024; Wang and Chang 2024) is not accompanied by a consensus in which trait combinations are likely to be most beneficial either overall or under specific drought regimes. We had too few genotypes in this study to do a robust comparison of drought tolerance by genotype, but it would be interesting to compare drought tolerance across genotypes that produce different combinations of high/low stomatal and vein densities under well-watered conditions, as well as differences in plasticity in these traits in response to drought, while simultaneously bringing other traits such as leaf size into consideration (see also below).

Looking at treatment responses for each trait individually, we found that the magnitude of relative plasticity for most traits increased as drought stress intensified. As expected, relative plasticity estimates were quite large for traits related to biomass production as well as traits related to leaf construction (e.g. secondary vein length per area, major vein length per area, leaf area, and leaf mass per area). In contrast, relative plasticity estimates for leaf anatomical traits such as measures of stomatal size, stomatal density, and minor vein length per area were much smaller—i.e. the degree of plasticity was smaller at the lower levels of organization. A similar trend has been documented in herbaceous species when subjected to other stress types. For example, in a study of plasticity in response to varying light intensities in rice (Chen *et al.* 2021), traits related to leaf morphology and anatomy exhibited less plasticity than growth-related traits. This pattern may result from the coordination of certain trait combinations that need to maintain specific relationships to ensure proper function—e.g. stomatal and vein traits exhibit strong correlations, the maintenance of which helps ensure efficient water use (Brodribb *et al.* 2013). In contrast, growth-related traits are perhaps more free to vary in response to changes in resource availability without impairing function.

### Trait covariation across and within treatments

#### Across treatments.

When combining data across treatments, trait responses co-varied such that we found several of the bivariate relationships expected based on the literature and their functional roles in plant-water relations (Doheny-Adams *et al.* 2012; Sack and Scoffoni 2013; Shahinnia *et al.* 2016; Fig. S2). Stomatal density and stomatal size were negatively correlated, consistent with the expectation of optimization of total area allocated to stomata (potentially through tradeoffs) affecting leaf level total stomatal conductance and thus total photosynthesis (Fiorin *et al.* 2016; Shahinnia *et al.* 2016; Bertolino *et al.* 2019; Harrison *et al.* 2020). Similarly, the positive association between stomatal density and minor vein length per area may represent a balance between stomata and veins such that water use and carbon acquisition are optimized (Brodribb *et al.* 2007; Sack and Scoffoni 2013; Carins Murphy *et al.* 2014). When viewing these trait relationships in multivariate space using PCA, trait differentiation among treatments was evident along major axes of variation dominated by plant size and stomatal traits.

#### Within treatments.

The overall structure of the bivariate correlation matrices was largely conserved across treatments, suggesting that the observed trait relationships were robust to environmental perturbations. However, looking within each treatment, the correlation structures were likely driven by the strong relationships among the size-related traits (total biomass, aboveground biomass, leaf area, and total number of stomata). The negative relationship between average stomatal density and average stomatal pore length that was significant across treatments, was not significant within treatments, but still showed a negative trend within each treatment. The positive relationship between average stomatal density and minor vein length per area that was significant across treatments was not significant or even consistently positive within treatments. Both of these relationships were previously observed across a much larger sampling of genotypes in a sunflower diversity panel grown under well watered-conditions (Earley *et al.* 2022), suggesting that the lack of trait correlations within treatments may have been due, at least in part, to either low sample sizes or ranges of variation too small to be able to detect significant patterns. However, an additional consideration is that the across-treatment relationships included both genetic differentiation among genotypes and phenotypic plasticity, whereas the within-treatment correlations were predominantly attributable to genetic differentiation—i.e. the scale of investigation matters.

### Relationship between trait variation and performance

Our results show that variation in just four leaf traits is strongly predictive (*R*^2^ = 0.74) of plant performance measured as total biomass production. These four traits (minor vein length per area, average stomatal pore length, average stomatal density, and leaf mass per area) were primarily chosen to reflect the major axes of variation identified in previous work () and documented again herein. This result suggests that these and/or closely related leaf traits are strongly associated with, and potentially impact overall plant performance, consistent with the role that veins and stomata play in gas exchange and water transport and thus photosynthetic capacity (Sack *et al.* 2013). However, establishing support for causal relationships between traits (e.g. anatomical, physiological, or life history) and growth or another proxy for fitness at an organizational level relevant to natural and artificial selection is challenging (e.g. Ackerly *et al.* 2000; Stanton *et al.* 2000; Shipley *et al.* 2006; Donovan *et al.* 2011; Mason *et al.* 2016; Caruso *et al.* 2020). We observed that the ways in which these traits are associated with biomass are environment-dependent. To make predictions about overall plant responses to diverse and complex stresses like drought, it is likely that trait complexes need to be considered in breeding efforts instead of a ‘one trait to rule them all’ approach.

As has been recognized previously, some of the coordination of leaf traits can be mediated through leaf size or epidermal cell size (e.g. Uhl and Mosbrugger 1999; Dunbar-Co *et al.* 2009; Sack *et al.* 2013; Carins Murphy *et al.* 2014, 2016; Wyka *et al.* 2019). While we did not set out to assess the impact of leaf size on drought response at any particular scale, evidence from the bivariate and multivariate assessments of trait relationships demonstrate that the extent to which leaf size mediated trait correlations depended on the scale at which we observed variation. Across treatments, which included a substantial amount of phenotypic plasticity, leaf area was negatively correlated with average stomatal density, average stomatal pore length, and minor vein length per area. As a result of this observation, we explored the influence of leaf area on the associations between our four focal traits and plant performance and found that we cannot entirely disentangle the effects of leaf area on the associations between our traits and biomass. Water limitation likely affected both cell division and cell expansion, and may have increased stomatal density and vein density in a coordinated manner through a passive effect since many cells and tissues scale allometrically in size (Brodribb *et al.* 2013; John *et al.* 2013). However, there is also evidence that vein density is not causally linked to epidermal cell expansion (Carins Murphy *et al.* 2016). Our results support the view that leaf size should be taken into account when considering drought effects on stomatal and vein traits, but that scaling with leaf size isn’t the whole story.

### Conclusion

Going forward, it will be important to further explore the extent of trait plasticity and trait correlations within and across treatments for cultivated sunflowers. There is genetic differentiation for a wide range of traits across cultivated sunflower lines, and thus potential for improvement through breeding (Mandel *et al.* 2013; Earley *et al.* 2022). Since the covariation of these traits is variable, decoupling plasticity in leaf anatomical and morphological traits might allow for the exploration of novel phenotypic space that could improve performance under drought and deserves further exploration.

## Supporting Information

The following additional information is available in the online version of this article –

Figure S1. Watering data based on percent water compared to field capacity—before and after watering each day. Dotted lines are target levels for each treatment: Well-watered (100%), Mild (60%), Moderate (40%), Severe (20%).

Table S1. Treatment means contrasts for all pairwise treatment comparisons following the ANOVA on ranked traits values reported in Table 2.

Table S2. Trait loadings (percentage of trait variation explained by each trait in the associated principal component [PC]) for Figs. 4A–D.

Table S3. Model summaries from our analysis of performance as a function of variation in Minor_VLA, SPL_Avg, SD_Avg, and LMA (natural log-transformed) across environments using a linear mixed effects regression model.

Table S4. Model summaries from our analysis of performance as a function of variation in Minor_VLA, SPL_Avg, SD_Avg, LMA (natural log-transformed), and Leaf_Area (natural log-transformed) across environments using a linear mixed effects regression model.

Table S5. Results from a model comparison comparing the multivariate regression on performance using four covariates as predictors (Minor_VLA, SD_Avg, SPL_Avg, and logLMA) without including Leaf_Area (presented in the table as mod_brm_1) with the same model in which logLeaf_Area was added as an additional covariate (presented in the table as mod_brm_2).

plae031_suppl_Supplementary_Materials

## Sources of Funding

This work was supported by a grant from the National Science Foundation Plant Genome Program (IOS-1444522) to JMB and LAD as well as funding from the International Consortium on Sunflower Genomics.

## Data Availability

Raw phenotypic data, underlying code, image, and model files are available via Dryad at https://doi.org/10.5061/dryad.xgxd254np.
