# Supplementary material for: Trait variation and performance across varying levels of drought stress in cultivated sunflower (Helianthus annuus L.)
Source: AoB Plants. 2024 May 27;16(4):plae031. doi: 10.1093/aobpla/plae031 (PMC11247526; doi:10.1093/aobpla/plae031)
Supplement: plae031_suppl_Supplementary_Materials [file plae031_suppl_supplementary_materials.pdf]

Trait variation and performance across varying levels of drought stress in cultivated sunflower (*Helianthus annuus* L.)

SUPPLEMENTAL FIGURES AND TABLES

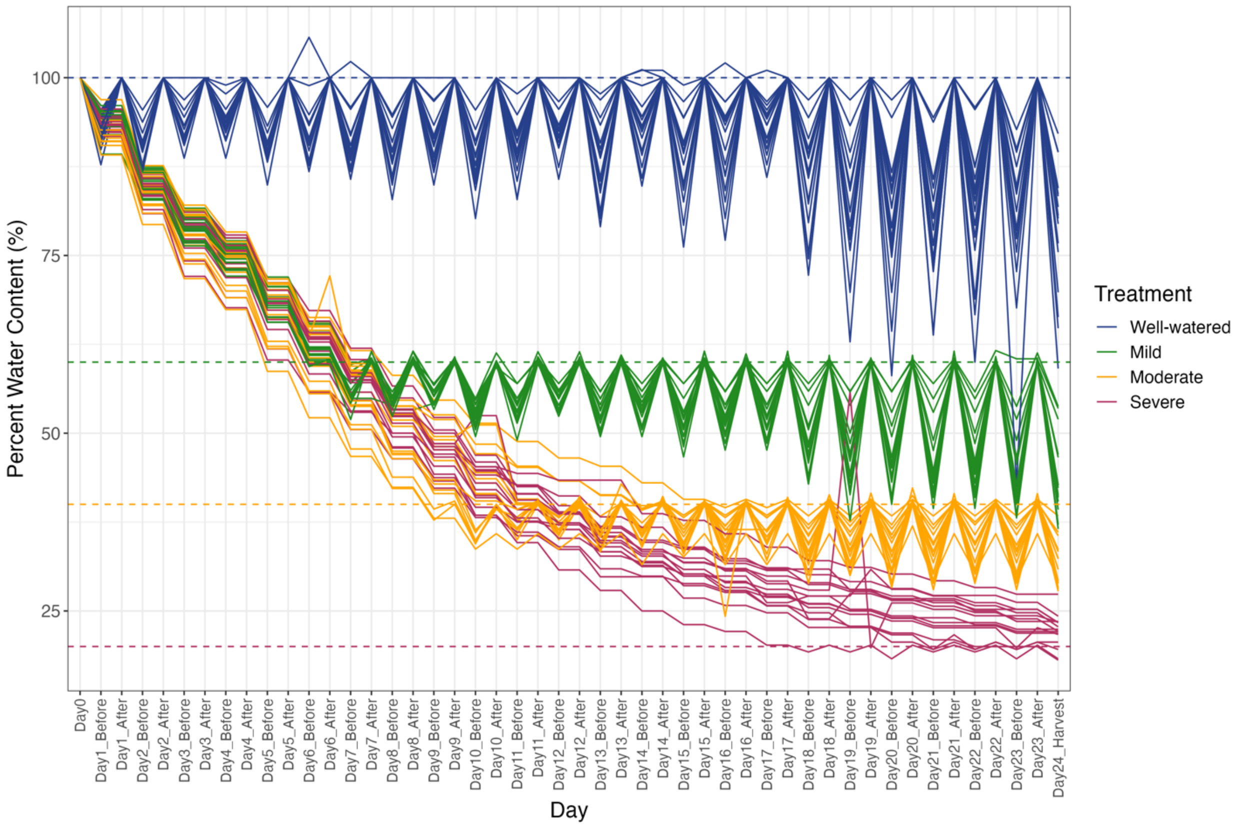

Figure S1: Watering data based on percent water compared to field capacity - before and after watering each day. Dotted lines are target levels for each treatment: Well-watered (100%), Mild (60%), Moderate (40%), Severe (20%).

Table S1: Treatment means contrasts for all pairwise treatment comparisons following the ANOVA on ranked trait values reported in Table 2. Significance for pairwise treatment differences indicated as (***P ≤ 0.001, **P ≤ 0.01, * P ≤ 0.05, P ≤ 0.1, ns = not significant). All traits are shown (trait abbreviations follow Table 1). Traits with no significant treatment effect reported from the ANOVA indicated with a dash (-).

	Well-Watered vs. Mild	Well-Watered vs. Moderate	Well-Watered vs. Severe	Mild vs. Moderate	Mild vs. Severe	Moderate vs. Severe
**AG_Bio (g)**	***	***	***	***	***	***
**Total_Bio (g)**	***	***	***	***	***	**
**Leaf_MF (g_{leaf}/g_{plant})**	*	*	**	ns	ns	ns
**Stem_MF (g_{stem}/g_{plant})**	-	-	-	-	-	-
**Root_MF (g_{root}/g_{plant})**	**	**	***	ns	*	*
**Leaf_Area (m^2)**	***	***	***	***	***	***
**LMA (g/m^2)**	ns	*	***	ns	**	#
**SD_Bot (stomata/mm^2)**	ns	ns	***	ns	**	*
**SD_Top (stomata/mm^2)**	ns	ns	**	ns	**	ns
**SD_Avg (stomata/mm^2)**	ns	ns	***	ns	**	#
**SPL_Bot (µm)**	ns	*	***	**	***	ns
**SPL_Top (µm)**	ns	*	***	#	***	*
**SPL_Avg (µm)**	ns	*	***	*	***	#
**SGCW_Bottom (µm)**	-	-	-	-	-	-
**SGCW_Top (µm)**	ns	ns	#	ns	ns	ns
**SGCW_Avg (µm)**	ns	ns	ns	ns	ns	ns
**Stomatal_Ratio (unitless)**	ns	ns	#	ns	ns	#
**Total_Stomata (count)**	***	***	***	***	***	***
**Minor_VLA (mm/mm^2)**	ns	***	***	*	***	**
**2nd_VLA (mm/mm^2)**	***	***	***	***	***	**
**Major_VLA (mm/mm^2)**	***	***	***	***	***	**
**SV (stomata/mm)**	-	-	-	-	-	-
**Midrib_Density (mg/cm^2)**	ns	**	***	#	***	ns
**Midrib_MF (g_{midrib}/g_{leaf})**	ns	**	***	#	***	*
**Chlorophyll (unitless)**	ns	ns	*	ns	ns	*
**gsmax (mol/m^2s)**	-	-	-	-	-	-

Table S2: Trait loadings (percentage of trait variation explained by each trait in the associated principal component [PC]) for Figures 4A (well-watered treatment), 4B (mild treatment), 4C (moderate treatment), and 4D (severe treatment). Trait abbreviations follow Table 1.

A) Well-watered Treatment

	PC1	PC2	PC3
**SD_Avg**	2.171	15.105	2.685
**Stomatal_Ratio**	0.060	3.284	5.367
**SGCW_Avg**	3.386	1.113	0.984
**SPL_Avg**	0.807	13.660	6.773
**Leaf_Area**	11.183	1.026	0.435
**LMA**	0.375	1.038	0.020
**Minor_VLA**	5.256	0.433	6.070
**Second_VLA**	10.700	0.087	1.311
**Major_VLA**	10.841	0.332	1.407
**SV**	0.013	19.063	9.807
**AG_Bio**	12.042	0.165	0.747
**Total_Bio**	11.788	0.660	0.804
**Midrib_Density**	6.108	3.000	2.359
**Midrib_MF**	2.584	9.026	15.492
**Chlorophyll**	0.109	0.542	11.613
**gsmax**	1.471	1.963	23.121
**Leaf_MF**	0.237	15.881	1.377
**Stem_MF**	6.729	1.273	7.069
**Root_MF**	4.783	12.088	0.230
**Total_Stomata**	9.359	0.261	2.330

B) Mild Treatment

	PC1	PC2	PC3
**SD_Avg**	0.654	10.493	11.655
**Stomatal_Ratio**	5.683	3.356	3.024
**SGCW_Avg**	0.980	3.066	15.587
**SPL_Avg**	0.456	0.223	35.634
**Leaf_Area**	10.353	0.135	0.586
**LMA**	0.425	12.031	1.259
**Minor_VLA**	5.631	4.728	4.521
**Second_VLA**	7.871	1.959	0.909
**Major_VLA**	8.133	1.473	1.452
**SV**	0.016	14.785	3.680
**AG_Bio**	10.090	0.018	0.262
**Total_Bio**	9.918	0.003	0.601
**Midrib_Density**	2.850	8.836	1.854
**Midrib_MF**	6.614	3.764	0.002
**Chlorophyll**	6.506	1.156	1.942
**gsmax**	0.689	13.258	1.764
**Leaf_MF**	0.113	11.834	7.725
**Stem_MF**	8.205	1.597	4.286
**Root_MF**	5.365	6.825	1.447
**Total_Stomata**	9.448	0.460	1.810

C) Moderate Treatment

	PC1	PC2	PC3
**SD_Avg**	0.098	20.698	3.376
**Stomatal_Ratio**	0.800	5.956	8.584
**SGCW_Avg**	2.253	9.688	2.411
**SPL_Avg**	4.895	14.611	0.402
**Leaf_Area**	10.754	0.203	0.106
**LMA**	4.060	0.824	5.122
**Minor_VLA**	2.450	9.941	6.497
**Second_VLA**	11.078	0.020	0.045
**Major_VLA**	11.493	0.341	0.175
**SV**	0.615	6.819	12.332
**AG_Bio**	9.809	0.734	0.189
**Total_Bio**	9.872	1.212	0.004
**Midrib_Density**	4.908	5.507	6.275
**Midrib_MF**	4.812	3.135	0.033
**Chlorophyll**	6.977	3.369	0.588
**gsmax**	2.059	8.669	5.924
**Leaf_MF**	0.472	0.626	22.900
**Stem_MF**	0.968	6.350	8.083
**Root_MF**	0.027	0.165	16.090
**Total_Stomata**	11.600	1.130	0.864

D) Moderate Treatment

	PC1	PC2	PC3
**SD_Avg**	11.056	0.046	0.748
**Stomatal_Ratio**	0.199	1.792	11.020
**SGCW_Avg**	3.279	4.957	7.575
**SPL_Avg**	5.896	0.200	0.937
**Leaf_Area**	3.227	7.683	5.347
**LMA**	0.154	18.176	0.040
**Minor_VLA**	4.713	4.005	8.820
**Second_VLA**	7.894	1.550	6.576
**Major_VLA**	7.820	1.647	6.901
**SV**	9.737	0.029	2.596
**AG_Bio**	8.399	5.223	0.830
**Total_Bio**	9.939	1.463	0.403
**Midrib_Density**	0.131	17.000	0.094
**Midrib_MF**	3.043	1.622	2.071
**Chlorophyll**	2.957	6.097	13.317
**gsmax**	9.414	0.904	3.656
**Leaf_MF**	0.002	12.244	13.202
**Stem_MF**	1.944	6.916	0.163
**Root_MF**	0.338	6.655	12.467
**Total_Stomata**	9.858	1.792	3.237

Table S3. Model summaries from our analysis of performance as a function of variation in Minor_VLA, SPL_Avg, SD_Avg, and LMA (natural log-transformed) across environments using a linear mixed effects regression model. Presented are the mean estimated association between each trait and Total_Bio (i.e., *B*, the mean of the posterior distribution) and in parentheses are the 95% credible intervals. The bracketed range reflects the 80% credible intervals.

	Model Estimates			
**Trait**	**Well-Watered**	**Mild**	**Moderate**	**Severe**
Minor_VLA	-0.64 (-1.05, -0.25)	-0.57 (-1.05, -0.08)	0.30 (-0.10, 0.72)	0.40 (-0.07, 0.86)
	[-0.91, -0.37]	[-0.88, -0.25]	[0.04, 0.57]	[0.10, 0.68]
SD_Avg	0.47 (-0.23, 1.17)	-0.22 (-0.90, 0.48)	0.49 (0.03, 0.98)	0.12 (-0.20, 0.43)
	[0.04, 0.92]	[-0.67, 0.24]	[0.19, 0.80]	[-0.08, 0.33]
SPL_Avg	-0.09 (-0.46, 0.30)	-0.48 (-1.09, 0.13)	0.62 (0.10, 1.15)	0.25 (-0.22, 0.72)
	[-0.33, 0.15]	[-0.86, -0.09]	[0.29, 0.96]	[-0.05, 0.55]
logLMA	0.70 (-0.22, 1.64)	0.02 (-0.77, 0.83)	-0.02 (-0.34, 0.30)	0.01 (-0.19, 0.20)
	[0.09, 1.28]	[-0.49, 0.56]	[-0.23, 0.19]	[-0.12, 0.13]
Total_Bio	1.49 (0.46, 2.65)	0.08 (-0.77, 1.16)	-0.40 (-1.18, 0.52)	-1.23 (-2.15, -0.27)
	[0.87, 2.15]	[-0.44, 0.62]	[-0.83, 0.05]	[-1.80, -0.70]

Table S4. Model summaries from our analysis of performance as a function of variation in Minor_VLA, SPL_Avg, SD_Avg, LMA (natural log-transformed), and Leaf_Area (natural log-transformed) across environments using a linear mixed effects regression model. Presented are the mean estimated association between each trait and Total_Bio (i.e., *B*, the mean of the posterior distribution) and in parentheses are the 95% credible intervals. The bracketed range reflects the 80% credible intervals.

	Model Estimates			
**Trait**	**Well-Watered**	**Mild**	**Moderate**	**Severe**
Minor_VLA	-0.28 (-0.53, -0.03)<br>[-0.44, -0.11]	0.11 (-0.48, 0.69)<br>[-0.28, 0.50]	0.08 (-0.17, 0.32)<br>[-0.08, 0.24]	-0.00 (-0.32, 0.32)<br>[-0.20, 0.20]
SD_Avg	0.46 (0.03, 0.86)<br>[0.19, 0.73]	-0.15 (-0.61, 0.33)<br>[-0.47, 0.15]	0.01 (-0.30, 0.32)<br>[-0.20, 0.21]	0.07 (-0.33, 0.48)<br>[-0.19, 0.34]
SPL_Avg	-0.09 (-0.32, 0.12)<br>[-0.24, 0.05]	-0.05 (-0.53, 0.45)<br>[-0.35, 0.27]	0.08 (-0.28, 0.46)<br>[-0.15, 0.32]	0.05 (-0.37, 0.45)<br>[-0.21, 0.31]
logLMA	0.47 (-0.10, 1.00)<br>[0.10, 0.82]	0.29 (-0.25, 0.84)<br>[-0.06, 0.63]	0.18 (-0.03, 0.37)<br>[0.05, 0.30]	0.06 (-0.14, 0.25)<br>[-0.07, 0.18]
logLeaf_Area	2.50 (1.89, 3.10)<br>[2.11, 2.89]	1.05 (0.51, 1.57)<br>[0.70, 1.38]	0.58 (0.28, 0.89)<br>[0.39, 0.78]	0.15 (-0.90, 1.18)<br>[-0.52, 0.81]
Total_Bio	-1.10 (-1.92, -0.30)<br>[-1.60, -0.60]	-0.12 (-0.55, 0.29)<br>[-0.36, 0.12]	-0.36 (-0.72, -0.03)<br>[-0.54, -0.19]	-0.81 (-1.94, 0.31)<br>[-1.54, -0.08]

Table S5. Results from a model comparison comparing the multivariate regression on performance using four covariates as predictors (Minor_VLA, SD_Avg, SPL_Avg, and logLMA) without including Leaf_Area (presented in the table as mod_brm_1) with the same model in which logLeaf_Area was added as an additional covariate (presented in the table as mod_brm_2). The comparison is a leave-one-out cross validation implemented using the function loo_compare() in the R package *loo* (Vehtari et al. 2017). The model that has the better predicted fit (ELPD) is presented first with the difference between this model and compared models reported as difference in ELPD (elpd_diff). A general rule is that we have strong evidence to "prefer" a model if the elpd_diff value between it and the lower model is greater than two times the standard error of that difference (se_diff). In this case, we have strong evidence that despite the increased model complexity in the model that includes logLeaf_Area, this model is a better fit than the model without. See https://mc-stan.org/loo/reference/loo_compare and references within for additional information.

	elpd_diff	se_diff
mod_brm_2	0.00	0.00
mod_brm_1	-26.30	6.20
